# Community perceptions of health and rodent-borne diseases along the Inter-Oceanic Highway in Madre de Dios, Peru

**DOI:** 10.1186/s12889-016-3420-3

**Published:** 2016-08-09

**Authors:** Gabriela Salmón-Mulanovich, Amy R. Powell, Stella M. Hartinger-Peña, Lara Schwarz, Daniel G. Bausch, Valerie A. Paz-Soldán

**Affiliations:** 1 Tulane University School of Public Health and Tropical Medicine, New Orleans, LA USA; 2 US Naval Medical Research Unit No. 6, Callao, Peru; 3 Universidad Peruana Cayetano Heredia, San Martín de Porres, Lima, Peru; 4 Swiss Tropical and Public Health Institute, Basel, Switzerland; 5 Mc Gill University, School of Environment, Montreal, QC Canada

**Keywords:** Rodent-borne diseases, Land use change, Qualitative methods, Longitudinal study

## Abstract

**Background:**

Madre de Dios is located in the southeastern Amazonian region of Peru. Rodents have been estimated to be the reservoirs for up to 50 % of emerging zoonotic pathogens, including a host of viruses, bacteria, and parasites. As part of a larger study involving both human and animal research, this study serves to obtain a broader understanding of the key challenges and concerns related to health and rodent-borne illnesses from the perspective of the people living in these communities.

**Methods:**

We used a mixed methods approach, which comprised of 12 focus group discussions, 34 key informant interviews and the application of a survey (*n* = 522) in four communities along the Inter-Oceanic Highway (IOH) in Madre de Dios, Peru over a two-year period.

**Results:**

Although 90 % of survey respondents answered that rodents can transmit diseases and had seen rodents in their homes and immediate surroundings, most could not name specific rodent-borne diseases and, when probed, described rodents as pests or nuisance animals, but were not concerned about acquiring illnesses from them. Key informant interview data suggests that there has been a perceived increase in the amount of rodents in the communities since the construction of the IOH, however this potential increase was not coupled with increased knowledge about diseases or perceived risks among these key informants. Health providers also mentioned a lack of diagnostic tools specific for rodent-borne illnesses. This may be related to the fact that although a common rodent-borne disease like leptospirosis is frequently detected in the region, it is not routinely and readily diagnosed, therefore the real burden of the disease and exposure risk can be underestimated. If rodent-borne diseases are not on the radar of health professionals, they may not consider presumptive treatment, which could result in unnecessary morbidity and mortality.

**Conclusion:**

Awareness of rodent-borne diseases is still lacking in the area, even among health care professionals within the communities, despite the known burden of diseases like leptospirosis. We expect to report further findings as we obtain more information from all the study components.

## Background

Rodents comprise a major part of virtually any ecosystem and are the most abundant class of living mammals, representing over 40 % of the total mammalian species [1]. In addition to the economic losses associated with rodents destroying crops and farms, they have been estimated to be the reservoirs for up to 30 % of emerging zoonotic pathogens, including a host of viruses, bacteria, and parasites [2]. Some of the more concerning rodent-borne pathogens in South America include *Hantavirus*, *Arenavirus*, *Leptospira*, and *Yersinia pestis*, the etiologic agents of hantavirus pulmonary syndrome (HPS), several haemorrhagic fevers, leptospirosis and plague, respectively [3, 4]. In 2011, four cases of HPS were reported in the Loreto region (northern Amazon basin) of Peru, and results from a study conducted in 1996 showed the presence of IgG antibodies to *Hantavirus* among 20 % of *Oligoryzomys microtis* rodents collected, a species prevalent in this region [5]. There are very few reported cases of rodent-borne diseases in Madre de Dios, a state [known as department in Peru] in the southern Amazon basin in Peru, where this study takes place. However, six out of 362 (1.2 %) rodents in this area were found to have evidence of *Hantavirus* antibodies (IgG) in their blood between October 2009 and October 2010 [6]. Surveillance information for both leptospirosis and HPS in this region is limited but research studies have found both *Leptospira* and *Hantavirus* antibodies in rodents in Madre de Dios and more specifically for the latter along the Inter-Oceanic Highway (IOH) [7].

Madre de Dios is extremely biodiverse and rich in natural resources, and is also home to people who face great economic and social hardships, such as the yearly flooding of some areas in the region and disease outbreaks [8–10]. Due to the ecological richness of the area, the people living in Madre de Dios struggle between the prospect of development and the increasing global need and pressure for conservation; battling between the fine balance of obtaining what they need to make a living from their environment, but not depleting it or contaminating it in an unsustainable manner. The region has undergone substantial development in recent years, including the construction of the IOH that traverses the region and has facilitated increased migration to the area [11, 12]. The changes in land-use associated with the construction of a highway and accompanying changes in migration can have significant impacts on the flora and fauna in the region [13]. This could be due to the construction of the highway itself, which causes habitat fragmentation or destruction [14]. It can also be due to the population boom that occurred in the region encouraged by the highway construction, and the increase in need for resources leading to the further destruction of the Amazonian rainforest.

This environmental change, forest fragmentation, associated with land-use change can also result in change in the microbial community, with the potential for shifting patterns of transmission of zoonotic pathogens to humans [15]. Past studies have shown that anthropogenically disturbed habitats are at the greatest risk for rodent-borne diseases [16]. However, disease transmission is not only dependent on environmental changes that may impact food availability and rodent population distribution, but also on social factors that place humans at higher risk of exposure (i.e., human living conditions, fine scale movement patterns, type of occupation, etc.). Bacteria such as *Leptospira* are transmitted through the contact of mucous membranes with infected urine or contaminated water, while certain viruses such as *Hantavirus* may enter the respiratory system from the aerosolization of rodent faeces [17]. Therefore, human living conditions, type of occupation, fine scale movement within their communities, and access to health care and treatment can significantly affect disease transmission dynamics [18, 19].

This study is part of a larger project investigating the effects of anthropogenic habitat perturbation and land-use change on rodent population dynamics and risk of rodent-borne diseases taking place in four communities located along the IOH. Therefore, the research focuses on the knowledge and perceptions of people living in communities along the IOH in Madre de Dios about their health and risk for rodent-borne diseases and simultaneously a team of veterinarians and field workers collected rodent samples using live traps in the four study communities. This allows identifying the rodent species living near these communities, as well as analysing rodent population changes and measure rodent-borne disease risk. Specifically, this paper reports the perceptions on health and rodent-borne diseases of the participants across the four study communities.

## Methods

### Study setting

**Fig. 1:**
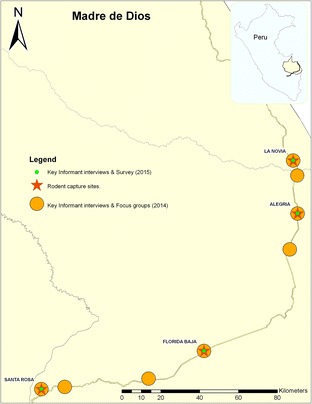
Map of the area

This data collection took place in two phases. The first phase was implemented in eight communities, ranging in size from 250 to 2500 residents based on numbers from the 2007 census data [15], that were located along the IOH in the Madre de Dios region of Peru (see Fig. 1) for map of the area). Half of the communities were northeast of Puerto Maldonado, the capital of Madre de Dios, and the other half of the communities were southwest. Key informant (KI) interviews and focus group (FG) discussions were conducted to assess general themes and issues regarding the community member’s health and well-being, as well as perceived risks, until saturation was reached.

In the second phase of data collection, in 2015, we collected data in only four of these communities: the communities selected for rodent trappings (every four months). We applied surveys to quantify topics we were interested in exploring based on initial findings of qualitative data collected in 2014. We also carried out additional KI interviews for more depth on the more important themes that had emerged in the first round of interviews in 2014. Half of these sites were located northeast, and half southwest, of the capital.

### Sampling, sample size, and analysis of human component

The communities were selected by convenience sampling based on community size, proximity to the IOH, accessibility (i.e., within a 3 h drive from Puerto Maldonado), and proximity to communities involved in a prior pilot study that aimed at exploring the impact of habitat perturbation on pathogen prevalence in reservoirs and vectors in the area surrounding the IOH [6].

#### Focus group discussions

We conducted twelve FGs with a total of 83 community members in eight communities in February 2014 (first phase). Purposive or convenience sampling was used to identify individuals for the FG, aiming for a range of individuals by age, as well as oversampling of residents who managed their household’s health and who had been living in the area for over five years. We stratified all but one of the FGs by gender because in our experience women do not participate as much in FGs when men are present, and because, in this region, women tend to be the ones who manage household illnesses. Also, since we were interested in the perspective of the person managing the family’s health, all of the women in the women’s only FGs were mothers. We were also interested in seeking historical perspective on life before and after the IOH, hence ten of the FGs were conducted with long-term residents exclusively. For all FGs, we recruited one to two days prior to scheduled FG dates through community authorities and our research team, but due to low attendance, we also recruited individuals who met the FG criteria on the streets the day of the FG.

Each FG was facilitated by a trained local anthropologist, and there were three note-takers whose detailed notes were compiled at the end of each day. A semi-structured FG guide was used for the FG discussions, focusing on the participants’ quality of life and health, as well as their perceptions of rodents in their communities and knowledge of rodent-borne diseases. Photos of two rodent species (*Oligoryzomys microtis* and *Neacomys spinosus*) common in the areas and that have been associated with HPS risk [5, 6] were shown to all FG participants; they were asked if they recognized or could identify the rodent species, and if so, where these were commonly found. FG data was summarized manually based on themes that emerged in the discussions. Findings are described in the text along with relevant quotes.

#### Key informant interviews

A total of 21 KI interviews were conducted with community leaders and healthcare personnel in eight communities in February 2014 (one to four KI interviews per community), and an additional 13 KI interviews were conducted in our four study sites in March 2015 - all identified by local collaborators at each site using convenience sampling. Questions were asked about the community, and changes observed in the communities in recent years. In 2014, the FG facilitator led these KI interviews, and detailed notes were taken by a notetaker. In 2015, a different trained researcher conducted the interviews, again with detailed notes taken by a notetaker. Audiotapes of all 2015 interviews were transcribed. KI interview data was summarized manually and thematically analysed – comparing and contrasting findings from the KI interviews with FG discussions and surveys. Themes have been described within the text and quotes used where relevant.

#### Survey

We applied a survey in 2015 in the four study communities. The survey comprised six sections--demographics, financial, personal, social, human, and physical capital--and was developed using themes that emerged from the 2014 FGs and KI interviews. We sampled all houses in each of these communities, conducting a total of 522 surveys. Based on the 2007 census information [20], we sampled 65 % of the houses. The most common reason for not applying surveys in some homes was that some were uninhabited at the time, likely a reflection of the seasonal migration in these communities. The surveys were applied by fieldworkers who received a two-week training, under careful supervision of a field coordinator. Survey data was entered and cleaned in Lima, and analysed using STATA 14.0 (StataCorp, College Station, TX).

#### Rodent trapping

The animal component of the study comprised the collection of rodent samples using live traps in six biological sampling grids (area of 70 m^2^). These were collected from areas with different disturbance levels (cattle grazing, pasture, crops, forest and border areas) near the four communities. Collections have been carried out every four months since December 2013 and are ongoing (until 2017). Details of these findings will be reported elsewhere, but a summary of relevant information from the first three trapping periods (December 2013, May and October 2014) is presented here.

#### Ethics

Approval for this study was obtained from the Institutional Review Boards of the US Naval Medical Research Unit No. 6 (NAMRU-6), the Universidad Peruana Cayetano Heredia, and Tulane University School of Public Health and Tropical Medicine. Community leaders and local authorities from the study area were informed of the study and agree to provide support. All participants - for the KI interviews, FG discussions, and surveys - provided written consent for their participation. For the rodent collection component, we had approval from the NAMRU-6 Institutional Animal Care and Use Committee and the Peruvian Forestry and Wildlife Service (SERFOR is the acronym in Spanish) by RD 0387-2012-AG-DGFFS/DGEFFS.

## Results

### Population characteristics

Household information gathered in the 2015 (census-like) survey revealed a young population (median age of 22) (Table 1). Approximately half (54.2 %) reported completing some secondary schooling. The main occupations reported were forestry (logging) and agriculture. The average household has 4.7 members.

**Table 1:** Characteristics of study participants

	Number	Percent	Mean (SD)
Sex			
Female	371	71.1	
Male	151	28.9	
Age	--	--	35.9 (12.9) range: 18–78
Level of education (informants)			
None	17	3.3	
Some primary (1–6 y)	169	32.4	
Some secondary (7–11 y)	283	54.2	
Some higher education (12+ y)	53	10.2	
Main occupation (household heads)			
Forestry	158	30.1	
Agriculture and farming	104	19.8	
Commerce	46	8.8	
Contracted labor	44	8.4	
Other	43	8.4	
Transportation	42	8	
Construction worker	39	7.4	
Housewife	19	3.6	
Health or education professional	8	1.5	
Government employee	3	0.6	
Administration	2	0.4	
Independent professional	2	0.4	
Fishery	1	0.2	
Unemployed/retired/NA	11	2.1	
Number of household members	--	--	4.7 (1.8)

### Knowledge of rodent-borne diseases and beliefs about transmission

In 2014, FG participants from all eight communities described the presence of rodents in their communities, especially around their houses and farms (see Table 2 for FG participants). When showed the pictures of two different rodent species, most FG participants recognized both the mice species (*Oligoryzomys microtis* and *Neacomys spinosus*). *O. microtis* was identified as the rodent more commonly found within the household. Most participants mainly described rodents as a nuisance, primarily because they eat the food in their houses, chew on their clothes and eat their crops and seeds.

**Table 2:** Composition of focus groups conducted in 2014

	Community size	Gender and number of participants per group		
Community code	(approx. # of households)	Female	Male	Mixed-gender
A	169	13	7	–
B	196	8	4	–
C	214	–	4	–
D	55	6	6	–
E	50	7	8	–
F	50	7	–	–
G	105	–	–	10
H	43	3	–	–
Total participants: 83		44	29	10

FG participants, however, had very little knowledge of diseases transmitted by rodents, and these diseases appeared to be of minimal concern to them. One woman in a community mentioned that rodents could spread fleas. Another woman from the same FG explained that she had read about cases of rodent-borne illness in Lima in the newspaper (likely referring to a recent case of HPS acquired in Iquitos, diagnosed in Lima). Participants from two communities mentioned that rodents transmit boils; a woman explained, “the boils fill with pus, grow for 15 days, burst and then disappear.” The participants of the male FG explained that diarrhoea could be transmitted by rodents. FG participants from other communities also mentioned an “itchy skin infection” transmitted by rodents. Although there were a variety of responses and descriptions of symptoms, most FG participants did not perceive rodents as transmitting diseases, they didn’t associate rodents with any severe health problems, and most of the mentioned problems were described as minor aggravations that do not have a major impact on their well being.

Information gathered from KIs in 2014 regarding rodents and rodent-borne diseases was similar to that of the FG participants (see Table 3 for KI characteristics). Only a couple of health care workers and an elected authority were aware of some health hazards associated with rodents. All of the KIs agreed that rodents are present in their communities, and several KIs from the health facilities specified that they had even seen rodents in their centers. When probed, the most commonly mentioned problem caused by rodents was the contamination of food. “Food contamination is very dangerous; people do not protect their food from the rodents,” explained the nurse from a community. Two nurses from different communities were aware that rodents are transmitters of certain diseases. One nurse mentioned leptospirosis as a health hazard associated with rodents, but also added that there have never been any reported cases. A nurse from a different community stated: “They transmit rabies and other infections, but we have not seen any cases.”

**Table 3:** Key informant data (2014/2015)

Community code	Year	Role	Number and gender
A	2014	Elected authority	4 male
B	2014	Health professional	1 female
		Elected authority	1 male
		Legal authority	1 male
	2015	Health professional	1 male
		Elected authority	1 male
		Educational professional	1 male
C	2014	Health professional	1 female
		Elected authority	1 male, 1 female
		Legal authority	1 male
D	2014	Health professional	1 female
		Elected authority	1 male, 1 female
	2015	Health professional	1 female
		Elected authority	1 male, 1 female
		Educator	1 male
E	2014	Elected authority	1 male
	2015	Educator	1 male, 2 female
F	2014	Health professional	1 female
		Elected authority	1 female
G	2014	Health professional	1 male
		Elected authority	1 male
	2015	Health professional	1 male
		Elected authority	1 male, 1 female
H	2014	Health professional	2 female

All of the KIs in health-related roles who were interviewed in 2015 had heard of at least one of the commonly known rodent-borne diseases when prompted (these included bubonic plague, HPS, leptospirosis, haemorrhagic fevers): “…we have heard of the diseases, we know they exist, but … there have been no reported cases [of those here]…. The people do not know of the existence of these diseases.” Less than half of the KIs with non-health related roles had heard of the diseases and, of those who had, bubonic plague was the most commonly recognized. Two KIs (one of whom was a medical professional and the other was an educational professional) also said, unprompted, that rodents are dangerous because they could spread rabies.

In two communities (B and E), none of the KIs thought that rodents were perceived as dangerous, whereas in other communities, KI representing a range of backgrounds, including health, revealed an accepted co-existence with rodents that was not perceived as very dangerous, though there was an acknowledgement that it might be due to lack of knowledge regarding possible diseases they transmit: “Because we don’t know what diseases or what they could do to us.” Or as another KI stated: “I’ve seen that the rodents here are healthy… I’ve seen what they eat: roots, fruit, Brazil nut, but there are few that are in the sewers…” Or, an example of the accepted coexistence: “…I think people are used to them, they don’t even kill them. Maybe people are not very aware [of diseases rodents transmit]”. Another KI from the health field reported that rodents were not a problem in the more “urban” parts of their community, but that people who work in the field harvesting maize get bitten and are at a higher risk: “Right here within the community I have not seen people complaining about rodents, but those who have complained are those who live outside of the town center… those who are outside have come to me because of rodent bites. It affects people who grow corn mainly.”

One KI (health professional) reported that there had been two cases of leptospirosis within the past year, with two farmers contracting the disease. The disease had been mistaken for dengue or malaria due to similarity in symptoms, and diagnosis had been difficult due to lack of diagnostic tools: “Yes… they have had leptospirosis… two cases last year. Farmers. People thought it was dengue…”. Aside from this information from this KI, there was no information available about incidence of any of the rodent borne diseases in these communities. Most of the KIs did not know about potential cases of rodent-borne diseases and three KIs (one medical professional, one educational professional and one elected leader) in three different communities were sure that there had not been any incidence of rodent-borne diseases.

Similar results were found in the survey applied in 2015: more than 80 % of all surveyed households had seen rodents in their house and over 65 % had seen them in their community. When asked if the rodents were considered a pest in the community, more than half (59 %) of the respondents answered yes, 33 % said no and 8 % did not know. Regarding rodent-borne illnesses, 90 % of the respondents from each community thought that rodents could transmit (unspecified) diseases. In accordance with KI and FG findings, survey participants responded that routes of infection for rodent-borne diseases were skin contact with rodent urine or faeces (26 %), contamination of the environment and food (26 %), and rodent bites (20 %). Only 2 % of respondents considered ectoparasites as vectors of infections (referred to as fleas or ticks within the survey). Of the 522 survey respondents, 65 specified that there were “other” routes of infection and 60 of these respondents cited rodent hair as a form of infection transmission.

### Rodent control practices

Various rodent eradication methods were mentioned in the FGs, but owning a cat was the most common method. Other rodent control practices mentioned include the use of traps and poisons, such as “Campeón” or “Racumín”. In one FG, some participants mentioned the use of a powder that is placed along paths of the rodents; however, other participants in the same group disagreed with its effectiveness, explaining that rodents ate the powder but did not die from it.

KIs in 2014 had similar responses to rodent control as the FG participants, and spoke of their own experiences. Cats were the most effective rodent control practice: “Having a cat works, they guard the house,” explained a KI from a community. However, others described frustration at being unable to eradicate rodents after many efforts, because they urinate in people’s homes: “I’m getting tired of washing my clothes every time. Poison doesn’t kill them, neither do cats.” One KI mentioned that there had been an increased demand for poison for rat control (this was not verified). In 2015 KIs were not prompted for rodent control practices, but approximately a third of the KIs spontaneously mentioned similar strategies as those mentioned in 2014; one KI who also owned a shop on the IOH reported that people shopping for rat poison, and a few KIs mentioned that many people own cats for rodent control.

Findings from the 2015 survey were similar to those obtained via qualitative methods. To prevent rodents from entering the house, 32 % of respondents said the most effective means was to keep the house tidy, and 31 % stated that keeping a cat prevented rodents from entering. To eradicate rodents, the most common survey answer was to put poison down (47 %), keep a cat (24 %) or set traps (14 %). Other preventative responses included manually rodent-proofing the house (“closing the house”). When asked about reactions to seeing a rodent (in the house or “*chacra*” (agricultural field)), 51 % of respondents reported that they would kill it, 22 % would “shoo it away” and 12 % would ignore it. Regarding reasons why rodents enter people’s homes, participants responded that food was the main reason (88 %), followed by “dirt” (8 %), as well as that rodents were looking for a nest or refuge.

### Changes in rodent presence since IOH construction

Although some FG participants reported expecting an escalation in rodent numbers in the area associated with migration and increased human population due to the highway, none of the FG participants reported observing any changes in the number of rodents since its construction. However, we noted a difference in the comments made in FGs (conducted in 2014), the KI interviews from 2014 (whose observations were similar to those of the community) and the KI interviews in 2015. More than half of the KIs interviewed in 2015 reported observing an increase in the number of rodents since the IOH construction, while in 2014 all KIs reported the presence of rodents, but only a few reported increased numbers. The main reason given by the KIs in 2015 for the increase in the number of rodents was the increase in the number of people, as well as more small stores and restaurants in the area: “Now that we are a number of people, there are rodents. Especially where there are restaurant services, that’s where you find them”.

In contrast, one of the authors (GSM, who has worked on projects around the IOH for over 8 years) also reported noting a difference in the amount of rodents collected (as part of the animal component of the study) in smaller communities like La Novia in contrast to a baseline study conducted in 2009, where the area was considered of high abundance and diversity in rodent populations (Razuri, unpublished data). The rodent collections performed since 2013 in this site have had very low yield and trap success rate, which may be related to the expansion of agriculture, in-migration and settlement of people in the community, changes that have been evident throughout the study period and will be further analysed in another paper.

### Summary of findings from the animal component

As part of the research activities, a group of trained field epidemiologists, veterinarians and field workers conducted trapping sessions in six grids within each of the four study communities. Results from the first three trapping periods, conducted in December 2013 and in May and October 2014 are presented in Table 4.

**Table 4:** Rodent species collected ^a^ in each of the study communities

	Study Community									
Species	Alegria	(%)	Florida Baja	(%)	La Novia	(%)	Santa Rosa	(%)	Total	(%)
*Euryoryzomys macconnelli*	1	(0.4)							1	(0.2)
*Euryoryzomys nitidus*	25	(11.1)	22	(10.6)	4	(15.4)	14	(10.4)	65	(10.9)
*Holochilus sciureus*	2	(0.9)	2	(1.0)			1	(0.7)	5	(0.8)
*Hylaeamys perenensis*	9	(4.0)	8	(3.8)	1	(3.8)	38	(28.4)	56	(9.4)
*Metachirus nudicaudatus*	3	(1.3)	1	(0.5)					4	(0.7)
*Neacomys spinosus*	25	(11.1)					3	(2.2)	28	(4.7)
*Necromys lenguarum*	27	(11.9)	34	(16.3)					61	(10.3)
*Oecomys bicolor*	10	(4.4)	15	(7.2)	2	(7.7)	13	(9.7)	40	(6.7)
*Oecomys roberti*							2	(1.5)	2	(0.3)
*Oligoryzomys bicolor*			2	(1.0)					2	(0.3)
*Oligoryzomys microtis*	89	(39.4)	109	(52.4)	10	(38.5)	50	(37.3)	258	(43.4)
*Oxymycterus inca*	22	(9.7)					1	(0.7)	23	(3.9)
*Proechimys pattoni*	8	(3.5)	4	(1.9)	7	(26.9)	2	(1.5)	21	(3.5)
*Proechimys simonsi*	4	(1.8)	10	(4.8)	1	(3.8)	9	(6.7)	24	(4.0)
*Proechimys sp*	1	(0.4)	1	(0.5)			1	(0.7)	3	(0.5)
*Rattus rattus*					1	(3.8)			1	(0.2)
	226		208		26		134		594	

^a^Collection period ranges from December 2013-October 2014, three trapping periods

These data show that *O. microtis*, *E. nitudus* and *N. lenguarum* were the most commonly trapped rodents in these study sites. Additionally, the type of habitat (undisturbed, edge and disturbed) where these species were most frequently found is in Table 5. As stated before, participants of the FGs in 2014 reported seeing *O. microtis* in their households, which have been found in more abundance than other rodent species throughout the four communities and mainly in edge and disturbed areas.

**Table 5:** Most common rodent species per disturbance gradient

Disturbance category	Non-disturbed		Edge		Disturbed		
Rodent species	N	(%)^a^	N	(%)^a^	N	(%)^a^	Total per species
*Oligoryzomys microtis*	3	(2.50)	250	(54.2)	71	(50.7)	324
*Euryoryzomys nitidus*	20	(16.7)	51	(11.1)	4	(2.9)	75
*Necromys lenguarum*	0	(0.0)	40	(8.7)	38	(27.1)	78
Total of 3 species	23	(19.2)	341	(74.0)	113	(80.7)	477
Total rodents collected	120		461		140		721

^a^ Collection period ranges from December 2013-January 2015, four trapping periods

## Discussion

Evidence of rodent-borne disease agents has been identified in rodents in the areas surrounding these communities, including antibodies to *Hantavirus* in *N. spinosus*, *N. lenguarum*, *E. nitidus* and the presence of the virus in two *N. spinosus* mice [6]. While there are several longitudinal studies examining rodent populations and their changing risk for disease transmission [21–24], including our larger study, this manuscript focuses on the human component of this study, specifically the perception of risk regarding rodent-borne diseases among the communities living in the area. Since the prevalence of rodent-borne diseases is not well known, it is unclear if these will become a health problem for people of the region, especially since species previously associated with diseases in this and other regions are prevalent in these communities. Noticeably, one of the most common species found via rodent trappings and identified by FG participants is *O. microtis* (43 %), which has been related before with *Hantavirus* in the Loreto department in the northern Amazon Basin [5]. Our findings in the animal component suggest that there is a differential distribution of some of the more frequent species (Tables 4 and 5). *O. microtis* has been found frequently in edge and disturbed areas, suggesting that the report of finding the rodents within the households may be feasible.

Based on our qualitative study, rodents were primarily described as a nuisance: they eat their food and can carry fleas and are a very common sight in local households. Residents of small communities along the IOH in Madre de Dios were not familiar with the risk of specific rodent-borne diseases or potential for exposure through their excreta or arthropods. However, exposure to urine or faeces was listed as the most common route of infection by survey respondents and the survey also showed that the communities were very aware that rodents carry diseases.

Due to the high burden reported in previous studies, it is mandatory to report leptospirosis in the region [25]. Recently, a sero-prevalence study in Puerto Maldonado revealed that 11.3 % of the population had been exposed to *Leptospira* (Salmon-Mulanovich, unpublished data). Nonetheless, few participants from our study could name any rodent-borne diseases. This limited knowledge about diseases is understandable considering that even if individuals in these communities have acquired rodent-borne diseases, these are likely misdiagnosed due to the lack of awareness of these diseases and the resource-limited health centers. For example, leptospirosis could easily be misdiagnosed for dengue, malaria or influenza, because of the similarity of the initial symptoms. Therefore, it is likely there are underreported cases of rodent-borne diseases in these communities due to the limited laboratory infrastructure and health care personnel within the communities.

Early diagnosis or treatment of more common rodent-borne diseases, such as leptospirosis, could reduce morbidity and mortality [26]. Surveillance information from this region could be used to guide clinical assessment of cases to the most prevalent diseases. It could also impact community confidence in health care worker practices. Research shows that junior doctors working in rural regions of Peru known as “serumistas”) find it hard to make diagnoses because they only have limited diagnostic tools available and limited support from senior clinicians [27]. In the case of rodent-borne diseases the diagnostic tests would have to be completed in laboratories and it has been found that people are reluctant to be referred for additional testing or specialist consultations due to financial constraints, money or the low likelihood of it being a serious problem [27]. This, in turn, can impact the relationship and confidence between healthcare providers and their patients.

Studies have shown that disturbed habitats are ideal for the emergence of rodent-borne diseases [19, 28]. Therefore, biodiversity loss caused by habitat perturbation from the construction of the IOH may have an effect on rodent-borne diseases considering the preference of some rodent species for edge and disturbed areas, the reported frequent contact of participants with rodents in this area, and that the main occupations of household heads in these communities are forestry and agriculture. Possible increased exposure to rodents through these occupations could present potential for increase in rodent-borne disease incidence in Madre de Dios [29–31]. Rodent trapping between 2013 and 2015 showed a lower yield and diversity of rodents in one study site compared to 2009, whilst qualitative findings showed that KIs had noticed an increase in the number of rodents. This data is meaningful and suggests that there may be an increase in the number of common rodents that live in urban or human settlement areas, such as *Rattus rattus* or *Mus musculus*, and a decrease in the diversity of other rodent species (wild rodents). At any rate, the data collected in the past two years shows a shift in the population composition of the different species of rodents depending on the level of disturbance of the land, probably as more generalist species take over habitat ranges previously used by specialists [19]. These changes will likely impact the disease transmission dynamics in this area similarly to what has been suggested in a previous study by Dizney *et al* where *Hantavirus* prevalence showed an increase in areas with decreased biodiversity [32].

By design, the sample size of the qualitative portion of the study was limited, but adequate for exploring the key themes we were interested in, and saturation in our communities was reached. Future research, both of human and rodent components, should expand to more communities along the IOH, as well as along rivers in the region, to allow for more rich and diverse comparisons, and to examine how rodent diversity and population size compares by type of setting and the varying land use in different communities. However, the results of this study have implications for future research and policy not only in Madre de Dios, but also for other geographically similar regions of the world [33–37]. Rodent-borne diseases are a class of diseases that do not have as high an incidence as other diseases like malaria, dengue, and leishmaniasis [13]; however, it is possible that they are more common than we think. Another limitation in the qualitative data collection was that different researchers collected data in 2014 and 2015. Whilst both followed a rigorous methodology, there may have been differences in questioning style and depth and type of answers gleaned; however, this may have also allowed for more diverse answers. Another limitation was the selection of study sites for the animal component. Urban areas such as households, schools, shops and restaurants were not included for rodent trappings. Hence, the design of the study will only allow us to identify changes in the restricted areas where the grids have been set and do not permit sampling in a wide variety of habitats in order to link participants’ observations within their households and the species of rodents collected. Between 2014 and 2015, the KI interviews showed an improved knowledge of rodent-borne disease. This may have been because of differences in questioning styles and more detailed questioning around knowledge of rodent-borne disease, or it could also have been due to presence of personnel from the on-going animal component of the study in the communities bringing attention to rodents and possible diseases they transmit. The increase in knowledge was most noticeable amongst healthcare professionals, which may indicate improved training amongst healthcare professionals, more awareness of the research teams or higher exposure to possible cases.

## Conclusions

In summary, we found that the general population are not aware of rodent-borne diseases and are not concerned about rodents or their presence; they are seen as pests. There are mixed reports regarding changes in rodent population since the construction of the IOH with the most recent KI data suggesting that there has been an increase in rodents in the communities. However, this potential increase has not changed beliefs or attitudes about rodent-borne disease. Moreover, a lack of knowledge about rodent-borne diseases, even among health personnel, still persists in this region.

Findings from this project could be used to improve awareness of, and possible diagnosis of, rodent-borne diseases among health professionals in the region, and educating the communities about rodent-borne diseases to improve preventive behaviors. With increased awareness among health professionals and communities, the importance of seeking lab diagnostics for potential rodent-borne diseases is emphasized, reducing severe disease or even mortality associated to these diseases. Health care professionals who are not aware of prevalence of rodent-borne diseases in their region may not consider presumptive treatment for these, which could result in unnecessary morbidity and mortality.

## Abbreviations

FG, focus groups; IOH, Inter-Oceanic Highway; KI, key informant; NAMRU-6, Naval Medical Research Unit No. 6; RD, directorate resolution; SERFOR, Servicio Forestal y de Fauna Silvestre (Peruvian Forestry and Wildlife Service); SERUMS, Servicio Rural y Urbano Marginal de Salud (Peruvian rural work for junior doctors)
